# Effects of Patient and Tumor Characteristics on Central Lymph Node Metastasis in Papillary Thyroid Cancer: A Guide for Selective Node Dissection

**DOI:** 10.34172/aim.2022.115

**Published:** 2022-11-01

**Authors:** Saygin Altiner, Ramazan Kozan, Ahmet Cihangir Emral, Ferit Taneri, Ahmet Karamercan

**Affiliations:** ^1^Department of General Surgery, University of Health Sciences, Ankara Training and Research Hospital, Ankara, Turkey; ^2^Department of General Surgery, Faculty of Medicine, Gazi University, Ankara, Turkey; ^3^Department of General Surgery, Sincan State Hospital, Ankara, Turkey

**Keywords:** Central lymph node, Central neck dissection, Papillary, Prophylactic, Thyroid cancer

## Abstract

**Background:**

Prophylactic central lymph node dissection (CLND) in papillary thyroid carcinoma (PTC) is still controversial. This study aimed to analyze the factors related to the patient and tumor characteristics affecting central lymph node metastasis (CLNM) in PTC patients and to evaluate the contribution of the results to shaping the surgical treatment algorithm.

**Methods:**

Two hundred and fifty-five PTC patients who underwent total thyroidectomy and CLND were evaluated retrospectively. Histopathology reports were examined to reveal tumor characteristics. The CLNM ratio and the relationship between CLNM with clinicopathological and demographic characteristics were analyzed.

**Results:**

The incidence of CLNM was 54.9% (95 CI%: 49−60.8). Male gender (*P*=0.027), age<45 years (*P*=0.016), tumor size≥9.5 mm (*P*<0.001), lymphovascular invasion (*P*<0001) and extracapsular invasion (*P*=0.007) were factors that increased the risk of metastasis. The follicular variant decreased the risk (*P*=0.010). There was no relationship between CLNM and focality (*P*=0.054). A low-to-moderate correlation was found between tumor diameter and the metastatic lymph node (MLN) number/total lymph node number ratio (r=0.396, *P*<0.001).

**Conclusion:**

A selective prophylactic CLND strategy can be applied in cN0 patients. As the tumor diameter increases in PTC, both the risk of CLNM and the number of MLN increase. Lymphovascular and extracapsular invasion are other factors that increase the risk. The follicular variant is associated with a lower risk of CLNM. Male patients who are under the age of 45 and have a tumor diameter of 9.5 mm or more are definite candidates for prophylactic CLND.

## Introduction

 Papillary thyroid carcinoma (PTC) is the most common malignant endocrine cancer in the world and has drawn attention with the rapid increase in its incidence over the last few decades.^1^ Even though its long-term prognosis is quite good, recurrence is the most important factor in increasing morbidity and mortality.^2^ Available studies have shown that lymph node metastasis is the most important factor affecting both the local recurrence risk and overall survival in patients with PTC.^2-5^

 Lymphatic metastasis usually occurs first in the central compartment and then in the lateral neck compartment. Thus, central lymph node metastasis (CLNM) is the most common involvement in PTC. Naturally, skip metastases that do not comply with this pattern can be observed.^2,3^ Due to this pattern of behavior, it is important to plan initial treatment well in terms of protecting PTC patients from local recurrence caused by lymphatic involvement and the resulting morbidities.

 The standard surgical treatment in PTC is total thyroidectomy or lobectomy depending on the tumor location and size. Today, a consensus has been reached on therapeutic neck dissection in patients with clinical cervical lymph node metastasis.^6-9^ Nonetheless, there are contradictory opinions about the necessity and indications of prophylactic central lymph node dissection (pCLND) in patients with no clinical cervical lymph node metastasis (cN0). Supporters of pCLND rely on correct staging, reduced local recurrence, increased disease-specific survival advantages, and safety.^1,2,9-11^ The reasons for those who have the opposite view are based on the fact that prophylactic dissection increases the risk of hypoparathyroidism and nerve injury and does not confer a survival advantage.^2,12,13^

 Factors affecting CLNM in PTC are among the issues that have gained higher importance in recent years. It has been reported that the CLNM rate varies between 26.4% and 63.2% in cN0 patients.^14,15^ These results have made it inevitable to investigate the issue of performing pCLND as a part of surgical treatment, particularly in the cN0 patient group. There are approaches that suggest performing routine CLND in all PTC patients.^2,11^ On the other hand, the view of adopting a selective approach instead of routine CLND in cN0 patients has gained increasing popularity.^1,5,8,14,16^

 Clinical and radiological identification of predisposing factors specific to node-negative patients will guide the selection of candidate patients for pCLND and the extent of initial surgical treatment. In this study, we aimed to analyze the factors related to the patient and tumor characteristics affecting CLNM in PTC patients and to evaluate the contribution of the results to shaping the surgical treatment algorithm.

## Materials and Methods

**Figure 1:**
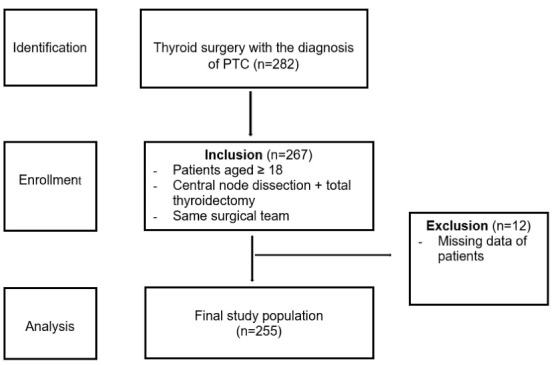
Overall Scheme of Sample Collection.

 Patients who underwent thyroidectomy with a diagnosis of PTC at the Department of General Surgery, Gazi University Hospital between September 2009 and September 2019 were identified with a retrospective analysis of the database. The diagnosis of PTC was based on ultrasound-guided fine-needle aspiration biopsy before surgery. Routine bilateral CLND was performed in all PTC patients. There was no patient with clinical or radiological lymph node metastasis prior to surgery. The inclusion criteria for the study consisted of several parameters: (1) being 18 years of age or older, (2) bilateral CLND with simultaneous total thyroidectomy, and (3) all surgical procedures performed by the same surgical team. The exclusion criteria were: (1) missing data on demographics, operation or pathology (Figure 1).

 Out of a total of 282 patients, 255 met the study criteria. Histopathology reports were evaluated to determine tumor characteristics. Patients’ age at diagnosis, gender, largest tumor diameter, tumor subtype, surgical margin, lobe where the tumor was located, localization within the lobe, focality, lymphovascular invasion, extracapsular invasion and CLNM were evaluated. Patients were further classified according to the Tumor-Node-Metastasis (TNM) classification. The classification was determined based on the 8th edition of the American Joint Committee on Cancer TNM staging system.

###  Statistical Analysis

 All statistical analyses were performed with the statistical software SPSS 20.0 (SPSS Inc., Chicago, IL, USA). The significance level for all analyses was considered as 0.05. Categorical measurements were presented as number and percentage, while continuous measurements were presented as mean and standard deviation, and also median and range. The compliance of the variables to normal distribution was evaluated by visual (histogram) and analytical methods (Kolmogorov-Smirnov/Shapiro-Wilk). Pearson’s chi-square test, Mann-Whitney U test, Spearman test, and receiver operating characteristic (ROC) curve analysis were used as statistical methods. Using ROC analysis, the predictive, sensitivity and specificity values were calculated. Cohen’s d index was used to assess the magnitude of the difference between CLNM positive and negative groups. Factors found to be significant in Pearson’s chi-square test were included in multivariable logistic regression analysis to identify the independent predictive factors.

## Results

 Sixty−one of the patients (23.9%) were men [95% confidence interval (CI) 18.8−29.4], and 194 (76.1%) were women (95% CI 70.6−81.2). The mean age was 38.4 ± 11.5 years (median 36, range 18−69 years). While 177 patients (69.4%) were under 45 years of age (95% CI 63.9−75.3), 78 patients (30.6%) were aged 45 years or older (95% CI 24.7−36.1). According to the TNM classification, 117 patients (45.8%) were categorized as T1a, 101 patients (39.6%) as T1b, 32 patients (12.6%) as T2, and 5 patients (2%) as T3. One hundred and forty patients (54.9%) had positive nodes (95% CI 49−60.8), whereas 115 patients (45.1%) had negative nodes (95% CI 39.2−51). There was no patient with T4 tumor or metastases to distant organs. According to the localization of the tumor in the thyroid gland, 98 patients (38.4%) (95% CI 31.4−43.9) had cancers in the right lobe, 89 patients (34.9%) (95% CI 28.6−40) had cancers in the left lobe, and 68 patients (26.7%) (95% CI 20.8−31.4) had cancers in both the right and left lobes. The distribution of the tumor throughout the thyroid lobe was determined. It was found in the superior lobe in 65 patients (27.5%) (95% CI 22−33.5), the middle lobe in 45 patients (19.1%) (95% CI 14.424.2), the inferior lobe in 59 patients (25%) (95% CI 19.5−30.1), the isthmus and isthmus-lobe junction in 40 patients (16.9%) (95% CI 12.3−22.4), and multilobarly and bilaterally in 27 patients (11.4%) (95% CI 7.6−15.7). In 19 patients (7.5%), no information could be obtained regarding the exact localization of the tumor (Table 1).

**Table 1:** Demographic and Tumor Characteristics of Patients

**Characteristics (n=255)**	**Number **	**Percent**
Gender		
Men	61	23.9%
Women	194	76.1%
Age at diagnosis (year)		
< 45	177	69.4%
≥ 45	78	30.6%
Tumor size (cm)		
≤ 1	117	45.8%
> 1 – ≤ 2	101	39.6%
> 2 – ≤ 4	32	12.6%
> 4	5	2%
Tumor subtype		
Classic	185	72.5%
Follicular	49	19.2%
Others	21	8.3%
Surgical margin		
Negative	231	90.6%
Positive	24	9.4%
Lobe with tumor		
Right	96	37.6%
Left	87	34.1%
Bilateral	66	25.9%
Isthmus	6	2.4%
Localization within the lobe		
Superior	65	27.5%
Middle	45	19.1%
Inferior	59	25.0%
Isthmus or junction	40	16.9%
Multiple	27	11.4%
Unknown	19	7.5%
Tumor focality		
Unifocal	161	63.1%
Multifocal	94	36.9%
Lymphovascular invasion		
Yes	150	58.8%
No	100	41.2%
Extracapsular invasion		
Yes	12	4.7%
No	243	95.3%
Lymphocytic thyroiditis		
Yes	129	50.6%
No	126	49.4%
Central node metastasis		
Yes	140	54.9%
No	115	45.1%

 Lymphovascular invasion was detected in 150 patients (58.8%) (95% CI 54−66.4), but not in 105 patients (41.2%) (95% CI 33.6−46). Histopathology reports were also evaluated with respect to the relationship between lymphocytic thyroiditis and PTC. Lymphocytic thyroiditis was detected in 129 (50.6%) of the patients (95% CI 71.5−84.2), but not in 126 of patients (49.4%) (95% CI 43.1−55.7). Extracapsular invasion was detected in 12 patients (4.7%) (95% CI 2.6−8.3), but not in 243 patients (95.3%) (95% CI 91.7−97.4) (Table 1).

 The mean diameter of the largest tumor was 13.6 ± 9.1 mm (median 11, range 1−60 mm) and the mean number of tumors was 1.7 ± 1.8 (median 1, range 1−21). The mean number of dissected lymph nodes was 9.1 ± 5 (median 7, range 4−26), whereas the mean number of metastatic lymph nodes (MLN) was 2 ± 2.9 (median 1, range 0−17).

 In univariate analysis, the risk of CLNM was shown to be higher in those under 45 years of age than those aged 45 years and over [odds ratio (OR) = 0.52, 95% CI 0.3−0.89, *P*= 0.016]. Additionally, male gender was associated an elevated risk of CLNM (OR = 1.97, 95% CI 1.13−3.72, *P*= 0.027). Other risk factors for CLNM were lymphovascular invasion (OR = 159.13, 95% CI 56.36−449.31, *P* < 0.001) and extracapsular invasion (OR = 10.04, 95% CI 1.27−79.07, *P*= 0.007). The relationship between histopathological subtypes of the tumor and CLNM was examined, and it was found that the follicular variant subtype had a lower probability of metastasis than the other subtypes (95% CI 0.09−0.29, *P*= 0.010). When the localization of the tumor in the thyroid lobe was evaluated, it was shown that tumors situated in the superior lobe had a lower probability of CLNM than tumors located in the other lobes (95% CI 0.25−0.44, *P* < 0.001) (Table 2). There was no relationship between CLNM and whether the tumor was unifocal or multifocal (OR = 1.67, 95% CI 0.01−0.23, *P*= 0.054). Multivariable analysis revealed that age at diagnosis (OR = 0.29, 95% CI -2.49− -0.29, *P*= 0.016), gender (OR = 1.22, 95% CI -0.99−1.55, *P*= 0.025), tumor subtype (OR = 0.44, 95% CI = -1.67− -0.21, *P*= 0.007), lymphovascular invasion (OR = 2.59, 95% CI 4.47–7.9, *P*= 0.001) and extracapsular invasion (OR = 11.17, 95% CI 1.15–20.53, *P*= 0.003) were statistically significant independent predictive factors. However, intra-thyroidal localization of the tumor was not significant for CLNM (OR = 1.64, 95% CI -0.17−1.09, *P*= 0.37) (Table 3).

**Table 2:** Factors Affecting Central Lymph Node Metastasis

**Factors**	**CLNM (-)**		**CLNM (+)**		***P*** ** Value**	**OR **	**95% CI**
	**n**	**%**	**n**	**%**			
Age group (n = 255)							
< 45 years	71	40.1%	106	59.9%	0.016	0.52	0.3–0.89
≥ 45 years	44	56.4%	34	43.6%			
χ^2^ = 5.808							
Gender (n = 255)							
Men	20	32.8%	41	67.2%	0.027	1.97	1.13–3.72
Women	95	49.0%	99	51.0%			
χ^2^ = 4.908							
Intra-thyroidal localization (n = 236)							
Superior	45	69.2%	20	30.8%	< 0.001	NA	0.25–0.44
Middle	22	48.9%	23	51.1%			
Inferior	13	22.0%	46	78.0%			
Isthmus/junction	15	37.5%	25	62.5%			
Multilobar	9	33.3%	18	66.7%			
χ^2^ = 30.705							
Tumor subtype (n = 255)							
Classic	80	43.2%	105	58.8%	0.010	NA	0.09–0.29
Follicular variant	30	61.2%	19	38.8%			
Others	5	23.8%	16	76.2%			
χ^2^ = 9.248							
Lymphovascular invasion (n = 255)							
No	99	94.3%	6	5.7%	< 0.001	159.13	56.36–449.31
Yes	16	10.7%	134	89.3%			
χ^2^ = 174,424							
Extracapsular invasion (n = 255)							
No	104	46.9%	129	53.1%	0.007	10.04	1.27–79.07
Yes	1	8.3%	11	91.7%			
χ^2^ = 6.874							

CLNM, central lymph node metastasis; OR, odds ratio; CI, confidence interval; NA, not applicable.

**Table 3:** Independent Predictive Factors of Central Lymph Node Metastasis in Multivariate Analysis

**Factors**	**OR**	**95% CI**	***P *** **Value**
Age at diagnosis	0.29	- 2.49 − - 0.29	0.016
Gender	1.22	- 0.99 − 1.55	0.025
Intra-thyroidal localization	1.64	- 0.17 − 1.09	0.37
Tumor subtype	0.44	- 1.67 − - 0.21	0.007
Lymphovascular invasion	2.59	4.47 – 7.9	0.001
Extracapsular invasion	11.17	1.15 – 20.53	0.003

OR, odds ratio; CI, confidence interval.

 The Mann-Whitney U test was used to determine the association between age and CLNM. The mean age of node-negative patients was 39 (range 2−68 years), while the mean age of node-positive patients was 34 (range 18−69) (*P* < 0.001). The mean tumor size in node-negative patients was 9 mm (range 1−35 mm), while it was 13 mm (range 3−60 mm) in node-positive patients (*P* < 0.001) (Table 4).

**Table 4:** Evaluation of Patients with and Without Central Lymph Node Metastasis in Terms of Age, Tumor Diameter and Lymph Node Status

**Patients **	**Node-Negative (Mean, Range)**	**Node-Positive (Mean, Range)**	**U value**	***P*** ** Value**	**Cohen’s Effect Size**
Age (y)	39 (31−68)	34 (18−69)	5993.5	< 0.001	0.38
Tumor diameter (mm)	9 (1−35)	13 (3−60)	4471.0	< 0.001	0.74
Multifocality	1 (1−5)	1 (1−21)	7016.5	0.040	0.30
Total lymph node number	6 (4−26)	8 (4−26)	6559.0	0.011	0.29
Metastatic lymph node number	0	3 (1−17)	0	< 0.001	1.67

**Figure 2:**
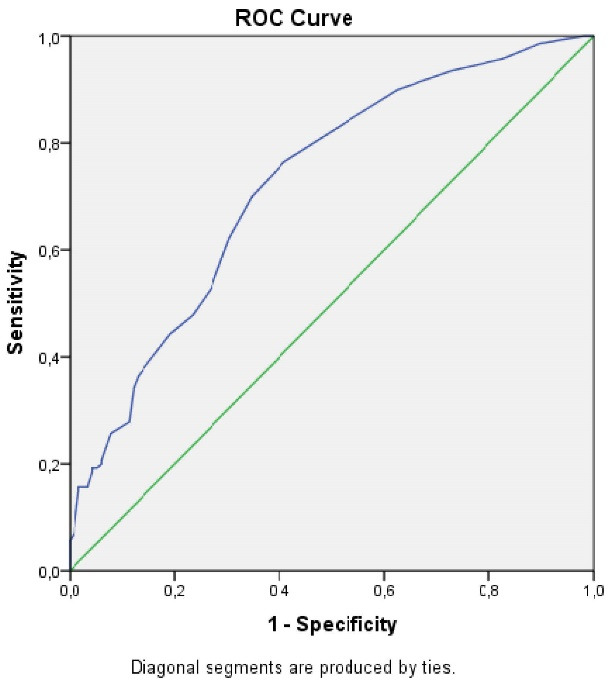
ROC Curve of the Largest Tumor Diameter in Terms of Central Lymph Node Metastasis.

 The correlation between tumor diameter and the ratio of MLN to total lymph nodes (TLN) was determined using the Spearman test. There was a low-to-moderate correlation between tumor diameter and the MLN/TLN ratio (r = 0.396, *P* < 0.001). A receiver operating characteristic (ROC) analysis was used to establish the cut-off value for the impact of tumor size on CLNM. As a result, the sensitivity of the largest tumor diameter greater than 9.5 mm in predicting CLNM was 76.4%, while the specificity was 54.1% (95% CI 0.66−0.79, *P* < 0.001) (Figure 2).

## Discussion

 Over the last two decades, the PTC incidence has increased dramatically.^1,5^ Patients with CLNM usually have poor outcomes, although their prognosis is better than that of other thyroid cancers.^4,5^ Due to the very high rate of regional lymph node metastases, there are divergent views on the extent of first surgical therapy for PTC patients. The discussion is on the trade-off between potential advantages of pCLND and postoperative complications.

 Patients with cN0 PTC who have advanced primary tumors (T3 or T4) or clinically involved lateral neck nodes (cN1b) should be considered for pCLND, according to the current American Thyroid Association (ATA) Management Guideline. The guideline recommends thyroidectomy for patients with small tumors (T1 or T2), as well as cN0 tumors, without performing pCLND.^17^ Nowadays, there are no discussion about performing routine neck dissection in the presence of a clinically positive cervical lymph node.^2,17^ It is remarkable that studies based on pathology results show that the rate of central compartment metastasis in cN0 patients can reach up to 63.2%.^15^ In our study, the rate of CLNM was found at 54.9%. Considering the effect of lymphatic metastasis on local recurrence and overall survival, the presence of CLNM in almost one out of two PTC patients may strengthen the recommendation for routine dissection. Support for this recommendation is that restaging after pCLND in patients initially staged as T1N0 changes the indications for radioiodine ablation in 30% of patients.^10,18^

 In patients with PTC who are scheduled for surgery, preoperative thorough neck ultrasonography (USG) assessment of regional lymph nodes is helpful for both prognosis and deciding the surgical approach. Here, the main problem is the low sensitivity of USG for detection of CLNM, unlike lateral cervical lymph node metastases.^19^ In a meta-analysis involving 18376 patients, it was reported that pCLND significantly decreased locoregional recurrence; however, it brought about higher complication rates.^20^ Although the central compartment is the most involved area, USG has poor performance in predicting this. It is essential to identify patients with high CLNM risk when these two aspects are considered together. This means that cN0 patients may be identified as candidates for pCLND, and the potential hazards of routine dissection can be reduced by using a selective dissection strategy.

 In our study, the relationship between the demographic characteristics of the patients and CLNM was investigated. Both age and gender factors were found to be effective. The risk of CLNM was found to be significantly higher in the group under 45 years of age compared to the group aged 45 and above. Similar results for the same age groups have been reported before in large patient series.^3,4,8,14,16,21,22^ Never the less, there are a few studies reporting that age and gender have no effect.^2,23^ Even though the female gender is defined as a risk factor for thyroid cancers, the male gender was found as a risk factor for CLNM in our study. The strong relationship between the male gender and lymph node metastasis cannot be ignored.^3,8,14-16,21,22^ Although the disease is more common in women, the higher rates of metastasis in the male gender have been explained by the differences in presenting to hospital and the tendency to present later in men.^24,25^ As a result of these findings, it would be appropriate to adopt a more skeptical clinical approach in the preoperative evaluation of cervical lymph nodes in male and young patients. It has been demonstrated that magnetic resonance imaging (MRI) is more sensitive than USG in detecting central compartment metastases in patients with PTC.^26^ MRI can guide pCLND as an option in the preoperative evaluation of patients in the high risk group.

 The findings on the link between PTC subtypes, focality, and intra-thyroidal tumor localization and CLNM are inconsistent. In our study, the follicular variant was found to reduce the risk of central compartment metastasis. Although its superiority over the classical variant was not presented statistically, it had been reported previously that the follicular variant was associated with a lower CLNM risk.^27^ However, the available evidence is inadequate for us to act with low metastasis expectation in patients whose preoperative histopathological examination is in favor of the follicular variant.

 When the localization of the tumor in the thyroid was examined, it was discovered that tumors located in the upper lobe had a lower risk of CLNM. On the other hand, there are studies reporting that the risk increases in tumors located in the middle and/or lower part of the thyroid lobe.^28,29^ It was also reported that the risk of metastasis increased in tumors in the isthmus, inferior anterior-central, inferior posterior-lateral, and middle posterior-lateral regions.^30^ It is clear that the results of studies examining the relationship between the intra-thyroidal tumor localization and central compartment metastasis do not agree with each other. As a result, it is inevitable to doubt the usefulness of location as a predictor of metastasis.

 Another characteristic investigated is the impact of focality. It has been reported that whether the tumor is unifocal or multifocal has no effect on the risk.^2,29^ On the other hand, there are studies reporting that multifocal PTC is predictive for CLNM, and there is a correlation between the number of tumor foci and the risk of metastasis.^5,14,23,31^ Our findings indicate that there is no correlation or relationship between focality and central compartment metastases. Additionally, lymphovascular and extracapsular invasions were found to be risk factors for CLNM. Nonetheless, as it is not possible to recognize these parameters in the preoperative period, it is clear that they cannot be used in patient selection for the selective dissection approach.

 One of the most important results of our study is the relationship observed between tumor size and CLNM. There are many studies indicate that the risk of CLNM increases in tumors of 10 mm and larger.^3,14,29,32^ However, the risk of metastasis has also been associated with much smaller tumor sizes.^8,16,21-23^ The cut-off value for our study is 9.5 mm, as determined by the risk-diameter association analysis. This indicates that even clinically T1N0 patients have a high risk of CLNM. Hence, the surgical treatment approach recommended by the ATA guideline for clinically T2N0 and PTC patients in the earlier stage should be investigated. A correlation between tumor size and both the metastasis risk and the number of MLN is another result of our study. Accordingly, tumor size is a prominent criterion in determining patients who will undergo pCLND.

 The most important limitation of our study is its retrospective nature. Additionally, it is a drawback that the preoperative radiological findings and clinical stages of the patients could not be matched with the pathological stages after CLND. It will be critical to research the correlation between the ultrasonographic and the pathologic tumor sizes in order to use the study results in clinical practice. Among the predictive factors identified in the study, those that are detectable only during the preoperative period have the potential to affect the surgical treatment protocol.

 In conclusion, a selective pCLND strategy can be applied in cN0 patients. Evaluation of predictive factors for central metastasis as a whole rather than using them alone will determine the high-risk patient group, for whom dissection will be inevitable. Male patients who are under the age of 45 and have a tumor diameter of 9.5 mm or more are definite candidates for pCLND.
