# Correction to "Effects of Patient and Tumor Characteristics on Central Lymph Node Metastasis in Papillary Thyroid Cancer: a Guide for Selective Node Dissection"

**DOI:** 10.34172/aim.31856

**Published:** 2024-08-01

**Authors:** Saygin Altiner, Ramazan Kozan, Ahmet Cihangir Emral, Ferit Taneri, Ahmet Karamercan

**Affiliations:** ^1^Department of General Surgery, University of Health Sciences, Ankara Training and Research Hospital, Ankara, Turkey; ^2^Department of General Surgery, Faculty of Medicine, Gazi University, Ankara, Turkey; ^3^Department of General Surgery, Sincan State Hospital, Ankara, Turkey

## Dear Editor,

 We have carefully reviewed the letter regarding our article titled “Effects of Patient and Tumor Characteristics on Central Lymph Node Metastasis in Papillary Thyroid Cancer: A Guide for Selective Node Dissection”.^1^ We sincerely appreciate the valuable comments and attention given by Shahraki HR.^2^

 We have re-examined the data set and statistical analyses retrospectively, and we also thoroughly reviewed all the analyses. We acknowledge that there were errors in transferring the results to the tables during the stage of presenting the outcomes associated with the conduct of univariate and multivariate analyses by different individuals. Additionally, we recognize that providing more detailed information in the tables would strengthen the comprehensibility of our study. Consequently, in the multiple logistic regression, we have reported odds ratios for individual categories by accepting one category as the baseline for quantitative variables with more than two categories.

 However, it is essential to emphasize that these corrections have not led to any changes in the findings and conclusions of our study.

 Below are the revised and detailed Table 2 and Table 3, reflecting the numerical changes in the results of univariate and multivariable analyses. The corrected paragraph in the “Results” section reads as follows:

**Table 2:** Factors Affecting Central Lymph Node Metastasis

**Factors**	**CLNM (-)**		**CLNM (+)**		***P*** ** Value**	**OR **	**95% CI**
	**n**	**%**	**n**	**%**			
Age group (n = 255)							
< 45 years	71	40.1%	106	59.9%	0.016	1.00	0.30-0.89
≥ 45 years	44	56.4%	34	43.6%		0.52	
χ^2^ = 5.808							
Sex (n = 255)							
Men	20	32.8%	41	67.2%	0.027	1.97	1.07-3.60
Women	95	49.0%	99	51.0%		1.00	
χ^2^ = 4.908							
Intra-thyroidal localization (n = 236)							
Superior	45	69.2%	20	30.8%	< 0.001	0.13	0.06-0.28
Middle	22	48.9%	23	51.1%		0.29	0.13-0.69
Inferior	13	22.0%	46	78.0%		1.00	
Isthmus/junction	15	37.5%	25	62.5%		0.47	0.19-1.14
Multilobar	9	33.3%	18	66.7%		0.56	0.21-1.55
χ^2^ = 30.705							
Tumor subtype (n = 255)							
Classic	80	43.2%	105	56.8%	0.010	1.00	
Follicular variant	30	61.2%	19	38.8%		0.48	0.25-0.92
Others	5	23.8%	16	76.2%		2.43	0.86-6.94
χ^2^ = 9.248							
Lymphovascular invasion (n = 255)							
No	99	94.3%	6	5.7%	< 0.001	1.00	52.20-365.83
Yes	16	10.7%	134	89.3%		138.19	
χ^2^ = 174,424							
Extracapsular invasion (n = 255)							
No	114	46.9%	129	53.1%	0.020	1.00	1.24-76.47
Yes	1	8.3%	11	91.7%		9.72	
χ^2^ = 6.874							

CLNM, central lymph node metastasis; OR, odds ratio; CI, confidence interval.

**Table 3:** Independent Predictive Factors of Central Lymph Node Metastasis in Multivariate Analysis

**Factors**	**OR**	**95% CI**	***P*** ** Value**
Age group		0.09-0.81	0.020
< 45 years	1.00		
≥ 45 years	0.27		
Sex		1.16-15.63	0.029
Men	4.26		
Women	1.00		
Intra-thyroidal localization			
Superior	0.39	0.09-1.66	0.204
Middle	0.81	0.17-3.85	0.790
Inferior	1.00		
Isthmus/junction	0.63	0.12-3.45	0.598
Multilober	1.17	0.20-6.74	0.861
Tumor subtype			
Classic	1.00		
Follicular variant	0.17	0.05-0.57	0.004
Others	0.58	0.06-5.33	0.634
Lymphovascular invasion			
No	1.00	65.55-1087.18	< 0.001
Yes	266.96		
Extracapsular invasion			
No	1.00	1.51-737.15	0.026
Yes	33.37		

OR, odds ratio; CI, confidence interval

 ‘In univariate analysis, the risk of CLNM was shown to be higher in those under 45 years of age than those aged 45 years and above [odds ratio (OR) = 0.52, 95% CI 0.30−0.89, *P* = 0.016]. Additionally, male gender was associated an elevated risk of CLNM (OR = 1.97, 95% CI 1.07−3.60, *P* = 0.027). Other risk factors for CLNM were lymphovascular invasion (OR = 138.19, 95% CI 52.20−365.83, *P* < 0.001) and extracapsular invasion (OR = 9.72, 95% CI 1.24−76.47, *P* = 0.020). The relationship between histopathological subtypes of the tumor and CLNM was examined, and it was found that the follicular variant subtype had a lower probability of metastasis than the other subtypes (95% CI 0.25−0.92, *P* = 0.010). When the localization of the tumor in the thyroid lobe was evaluated, it was shown that tumors situated in the superior lobe had a lower probability of CLNM than tumors located in the other lobes (95% CI 0.06−0.28, *P* < 0.001) (Table 2). There was no relationship between CLNM and whether the tumor was unifocal or multifocal (OR = 1.67, 95% CI 0.99−2.80, *P* = 0.054). Multivariable analysis revealed that age at diagnosis (OR = 0.27, 95% CI 0.09−0.81, *P* = 0.020), gender (OR = 4.26, 95% CI 1.16−15.63, *P* = 0.029), tumor subtype for follicular variant (OR = 0.17, 95% CI 0.05−0.57, *P* = 0.004), lymphovascular invasion (OR = 266.96, 95% CI 65.55−1087.18, *P* < 0.001) and extracapsular invasion (OR = 33.37, 95% CI 1.51–737.15, *P* = 0.026) were statistically significant independent predictive factors. However, intra-thyroidal localization of the tumor was not significant for CLNM (Table 3).’

 In conclusion, we believe that the valuable critiques and suggestions provided have enhanced the statistical transparency and accuracy of our study. We recognize the significance of reader engagement in research publications and how it contributes to the advancement of published studies.
